# Confirming Legionnaires’ Disease Outbreak by Genome-Based Method, Germany, 2012

**DOI:** 10.3201/eid2207.151738

**Published:** 2016-07

**Authors:** Florian Burckhardt, Andre Brion, Jeanette Lahm, Heinz-Ulrich Koch, Karola Prior, Markus Petzold, Dag Harmsen, Christian Lück

**Affiliations:** Federal State Agency for Consumer & Health Protection Rhineland-Palatinate, Landau, Germany (F. Burckhardt, A. Brion);; Local Health Authority Südwestpfalz, Pirmasens, Germany (J. Lahm, H.-U. Koch);; University of Münster, Münster, Germany (K. Prior, D. Harmsen);; Dresden University of Technology, Dresden, Germany (M. Petzold, C. Lück)

**Keywords:** outbreaks, Legionnaires’ disease, cooling tower, monoclonal antibody typing, Legionella pneumophila, multilocus sequence typing, whole genome sequencing, bacteria, respiratory disease

**To the Editor:** We report an outbreak of Legionnaires’ disease in southwestern Germany. On July 31, 2012, the State Health Agency of Rhineland-Palatinate was informed by the local health department of the city of Zweibrücken that 10 patients tested positive for *Legionella pneumophila*, the bacterium that causes Legionnaires’ disease. The onset of disease for all case-patients was from June 26 through July 25, which exceeded the yearly average of 1–4 patients a month. By August 23, we had received notifications of 19 patients with pneumonia and notification of 1 patient who did not exhibit pneumonia. We set 3 parameters for reporting a patient as a Legionnaires’ disease case-patient. First, the patient had to either live in or have been visiting the city of Zweibrücken in June 2012 before onset of disease. Second, the respiratory samples from the patient had to contain *L. pneumophila* or the results of patient’s serogroup 1 urinary antigen test had to be positive for the bacterium (*1*). Finally, clinical or radiologic confirmation of the disease was required. Of 20 patients who fit the case definition, 14 were male and 6 were female. Nine smoked and 2 were immunocompromised; none died.

All case-patients were positive for *L. pneumophila* serogroup 1 urinary antigen. From clinical samples of 2 patients, legionellae were cultured, and the infecting strain was confirmed as *L. pneumophila* serogroup 1, monoclonal subgroup Allentown-France, sequence type (ST) 82 (*2*,*3*). Currently, 118 strains of this ST are found in the European database for sequence-based typing of *L. pneumophila* (*2*). Most ST82 strains were isolated from clinical samples; thus, this ST appears more likely than other strains to infect humans. Further, 3 respiratory samples from case-patients were positive in a PCR for *L. pneumophila* serogroup 1 (*4*) but were negative by culture. These samples were investigated with the nested sequence-based typing protocol, which allows typing data to be obtained directly from clinical samples (*2*). Of the 3 samples, 2 were confirmed as ST82.

The local health authority did not initially identify likely sources of transmission such as cooling towers, public spas, or warm water supply systems in the vicinity of the patients (*5*). Environmental samples were taken from the homes of 15 of the 20 patients; all samples tested negative for *Legionella* (*6*).

To find the source of the outbreak, we plotted 20 home and 7 work addresses of patients using Quantum-GIS software (*7*) and found that 18 addresses were within a 2-km radius of each other, including 2 patients who had limited mobility and had not left their homes during their incubation period (Figure). We conducted a site visit on August 22 to inspect a sewage plant and 2 large manufacturing plants (A and B) that were within the same 2-km radius. Neither the sewage plant nor plant A had a potential *Legionella* source. Plant B had a cooling tower mounted on a rooftop that was described by the company as a closed circuit cooling system, indicating that no aerosols would be released, and thus was missed by the initial local health department inquiry. However, closed circuit referred only to the primary cooling circuitry, whereas excess heat was exchanged through wet surface cooling, allowing release of aerosols into the atmosphere. The local health department immediately shut down the cooling tower, and plant B used shot-dose chlorine to disinfect it. Before disinfection, we obtained 3 swab specimens and 250-mL samples of water from the reservoir and plated them in dilutions with and without acid wash (*6*,*8*). Samples without acid wash were completely overgrown, whereas a single 1-mL sample with acid wash showed 20 *Legionella* colonies after 7 days. Three colonies were typed and found to belong to the epidemic strain. Of the 27 work and home addresses, 6 were within a 1-km radius of the cooling tower, and 18 were within a 4-km radius (Figure). No further cases occurred within the incubation period (up to 14 days after closure of the cooling tower).

**Figure:**
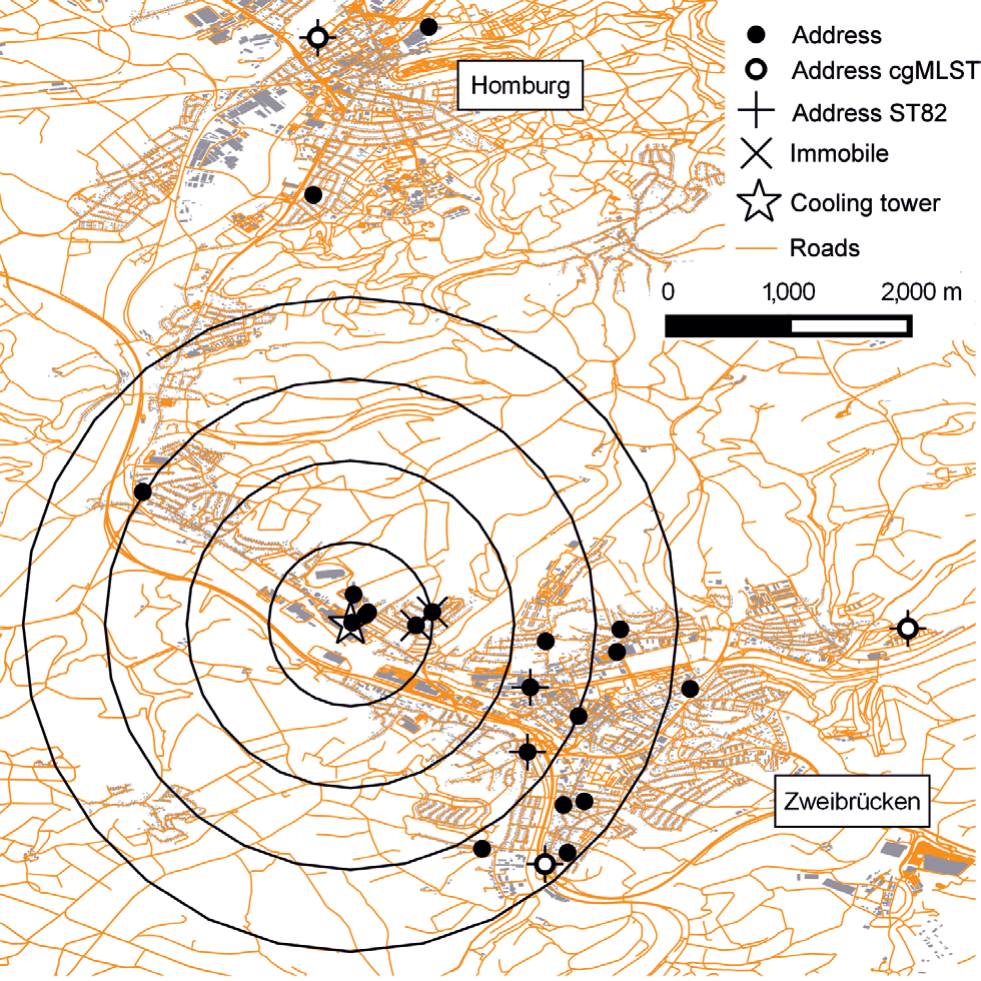
Geographic distribution of cooling tower and home and work addresses (n = 23) of patients; 1 patient may be represented twice with home and work address, because place of infection is unknown. The addresses marked “immobile” belong to 2 patients who had not left their homes. Two samples had undergone core genome multilocus sequence typing (cgMLST), and sequence type (ST) 82 was typed (represented by 2 home addresses and 1 work address). For 2 samples, only ST82 was typed. Two dots in the 1-km radius are overlapping each other. Four addresses (9 km, 10 km, 19 km, and 26 km from the cooling tower) are outside the scale of the map. Circle radii are from 1 km to 4 km, centered on the cooling tower. Shapefiles for mapping by OpenStreetMap contributors.

To further confirm this cooling tower as the source of the outbreak, we applied core genome multilocus sequence typing (cgMLST) (*3*). We analyzed allelic differences of 1,521 gene targets of the core genome of *L. pneumophila* using the pairwise ignore missing values option in SeqSphere+ software (Ridom GmbH, Münster, Germany). Results showed that the strains from 2 patients with culture-positive test results and the 3 environmental ST82 strains were identical in their cgMLST profile, which covers 47% of the Philadelphia-1 reference genome.

Currently, no German law requires a registry for cooling towers; such a registry would accelerate identification of potential *L. pneumophila* emission during outbreaks (*9*). In January 2015, a code of conduct for maintenance of cooling towers went into effect (*10*). Modern typing methods such as cgMLST can serve as supporting tools in confirming infection origin. However, this method must be validated on a larger scale, and its discriminatory power compared with that of current typing methods. Further cgMLST studies with other ST82 strains are underway.
